# Comparative analysis of dimethyl fumarate and fingolimod in relapsing–remitting multiple sclerosis

**DOI:** 10.1007/s00415-020-10226-6

**Published:** 2020-09-24

**Authors:** Johannes Lorscheider, Pascal Benkert, Carmen Lienert, Peter Hänni, Tobias Derfuss, Jens Kuhle, Ludwig Kappos, Özgür Yaldizli

**Affiliations:** 1 Neurologic Clinic and Policlinic, Departments of Medicine, Biomedicine and Clinical Research, University Hospital Basel, University of Basel, Basel, Switzerland; 2 Clinical Trial Unit, Department of Clinical Research, University Hospital Basel, Basel, Switzerland; 3 Rheinburg Klinikum, Walzenhausen, Switzerland; 4 Swiss Federation for Common Tasks of Health Insurances (SVK), Solothurn, Switzerland

**Keywords:** Dimethyl fumarate, Fingolimod, Effectiveness, Relapsing–remitting, Multiple sclerosis

## Abstract

**Background:**

Dimethyl fumarate and fingolimod are oral disease modifying treatments (DMTs) that reduce relapse activity and slow disability worsening in relapsing–remitting multiple sclerosis (RRMS).

**Objective:**

To compare the effectiveness of dimethyl fumarate and fingolimod in a real-world setting, where both agents are licensed as a first-line DMT for the treatment of RRMS.

**Methods:**

We identified patients with RRMS commencing dimethyl fumarate or fingolimod in the Swiss Federation for Common Tasks of Health Insurances (SVK) Registry between August 2014 and July 2019. Propensity score-matching was applied to select subpopulations with comparable baseline characteristics. Relapses and disability outcomes were compared in paired, pairwise-censored analyses.

**Results:**

Of the 2113 included patients, 1922 were matched (dimethyl fumarate, *n* = 961; fingolimod, *n* = 961). Relapse rates did not differ between the groups (incident rate ratio 1.0, 95%CI 0.8–1.2, *p* = 0.86). Moreover, no difference in the hazard of 1-year confirmed disability worsening (hazard ratio [HR] 0.9; 95%CI 0.6–1.6; *p* = 0.80) or disability improvement (HR 0.9; 95%CI 0.6–1.2; *p* = 0.40) was detected. These findings were consistent both for treatment-naïve patients and patients switching from another DMT.

**Conclusion:**

Dimethyl fumarate and fingolimod have comparable effectiveness regarding reduction of relapses and disability worsening in RRMS.

**Electronic supplementary material:**

The online version of this article (10.1007/s00415-020-10226-6) contains supplementary material, which is available to authorized users.

## Introduction

Dimethyl fumarate and fingolimod are oral drugs, which are approved as first-line disease modifying treatment (DMT) for patients with relapsing–remitting multiple sclerosis (RRMS) in Switzerland [1, 2]. Both agents effectively reduce relapse activity and delay short-term disability worsening in patients with RRMS compared to placebo [3–7]. While the results from the pivotal phase III studies suggested a similar efficacy of dimethyl fumarate and fingolimod compared to placebo, a direct comparison with a head-to-head randomised controlled trial (RCT) has not been performed, yet. A matching-adjusted study of pooled data from four phase III trials found comparable effects on relapses and disability outcomes of the two compounds [8]. However, RCTs have specific inclusion and exclusion criteria and are hence of limited generalizability. An alternative strategy is the use of observational data with appropriate statistical methodology [9, 10]. To date, a few observational studies have compared the real-world effectiveness of dimethyl fumarate and fingolimod with conflicting results: While several studies demonstrated a similar effectiveness of both drugs on relapse rates [11–15], a recent a study from the international MSBase registry found that fingolimod was superior to dimethyl fumarate in suppressing relapses [16], and a multicentre study from Italy showed that fingolimod-treatment resulted in a lower risk of relapses and disability worsening compared to dimethyl fumarate in patients who had switched from a platform injectable drug [17].

Hence, our aim was to compare the effectiveness of dimethyl fumarate and fingolimod in different treatment scenarios in a real-world setting, in which regulatory requirements are identical for both compounds.

## Methods

### Database

Data were derived from the registry of the Swiss Federation for Common Tasks of Health Insurances (SVK) that controls reimbursement for DMTs in Switzerland [18] The SVK registry contains data from 15,552 patients who started DMTs between February 1995 and July 2019. Individual patient data are provided annually by board-certified neurologists and recorded in a prospective manner as part of the routine centralised reimbursement approval process. The data included standardised information on disease type, date of disease onset, relapse rate, and neurological status assessed using the Expanded Disability Status Scale (EDSS). The case record forms were checked for completeness and internal plausibility by a data manager at the SVK and entered into a database. Queries about missing or inconsistent data were issued by the SVK under the supervision of an independent physician, who was responsible for reimbursement decisions. The application for reimbursement was valid for 1 year. Patients were followed-up until they discontinued treatment or changed health insurance to a company that was not associated to the SVK. From 1995 to 2008 about 85% of all health insurance companies in Switzerland used the SVK forms for DMT reimbursement. This number decreased to about 65% in the year 2013 [19]. Written informed consent was obtained from all patients in accordance with the Declaration of Helsinki. The Ethics Committee of North-West and Central Switzerland confirmed that this study does not fall under the Swiss Federal Act on Research involving Human Beings, due to the administrative nature of the data and adequate anonymisation (Req-2019-00470).

### Study population

For this analysis, we selected patients with RRMS who started or switched to treatment with dimethyl fumarate or fingolimod. The minimum required dataset consisted of complete data for MS disease type, date of disease onset, date of last relapse, the EDSS and Functional System (FS) scores and at least one subsequent on-treatment visit. For the analysis of 12-months confirmed disability worsening and disability improvement, at least two annual follow-up evaluations were required. To ensure the validity of the matching process, we restricted the analysis to patients who switched DMTs after August 2014 when dimethyl fumarate became available in Switzerland and excluded patients switching from fingolimod to dimethyl fumarate or vice versa [2].

### Study endpoints

The primary study endpoints were the time to first relapse and annualised relapse rate (ARR). ARR was calculated as the annualised number of relapses between treatment initiation and censoring. As secondary study endpoints, we used disability worsening, which was defined as an increase of ≥ 1.5 points from an EDSS score of 0.0, ≥ 1.0 point from an EDSS score of 1.0–5.5 or ≥ 0.5 point from an EDSS score ≥ 6.0 confirmed in the following year and disability improvement, which was defined as a decrease of ≥ 1 EDSS step (≥ 1.5 points if baseline EDSS was ≤ 1.5 and ≥ 0.5 points if baseline EDSS was ≥ 6.0) also confirmed after at least 1 year [20]. Disease duration was calculated from the first demyelinating event.

### Matching and statistical analysis

Matching and statistical analysis was conducted by P.B. using R [21]. The included patients were matched on their propensity for receiving dimethyl fumarate vs. fingolimod using the MatchIt package [22]. The propensity score was based on a multivariable logistic regression model with treatment allocation as the outcome variable and the demographic and clinical variables available to treating neurologists at the time of the treatment decision as the independent variables. These comprised age, gender, disease duration, baseline EDSS and FS scores, EDSS change in the preceding year, number of previous DMTs, time on first-line DMT, time since last visit, time since last relapse, number of relapses in the year before treatment switch and region in Switzerland.

Patients were matched in a 1:1 ratio using nearest neighbour matching within a calliper of 0.2 standard deviations of the propensity score. Patients who switched or discontinued treatment were censored. The pairwise on-treatment follow-up was determined as the shorter of the two individual follow-up periods for each matched patient pair (pairwise censoring) to control for attrition bias [10].

Time to relapse, EDSS worsening and improvement as well as treatment persistence were explored using Kaplan–Meier plots and analysed using marginal proportional hazards models, the cluster term indicating the matched patient pairs. The proportional hazards assumption was examined visually and by testing Schoenfeld’s residuals. After assessing the data distribution, the annualised relapse rate was estimated using a negative binomial regression model with the log of the follow-up time as offset variable.

The primary analysis included all eligible subjects, whereas the two secondary analyses were restricted to patients (A) on first-line and (B) on second-line treatment. In addition, we performed three sensitivity analyses: (1) matching with a calliper of 0.1, (2) omitting pairwise censoring and (3) also including patients starting fingolimod before August 2014.

Observed differences were considered significant if two-tailed *p* ≤ 0.05.

## Results

**Fig. 1:**
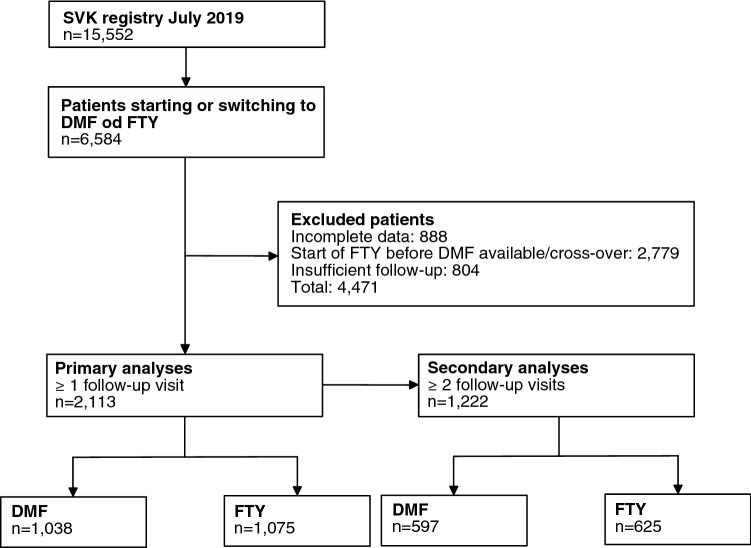
CONSORT flowchart of patient disposition. *DMF* dimethyl fumarate, *FTY* fingolimod

We identified 2113 eligible patients treated with dimethyl fumarate or fingolimod. Of these, 1222 (58%) had sufficient follow up to analyse the secondary endpoints (Fig. 1). Baseline characteristics of the cohorts are shown in Table 1 and in Supplementary Table 1 for the dataset used to analyse disability outcomes. Several demographic factors and markers of disease activity differed between the unmatched patient groups. The logistic model used to estimate the propensity scores showed that fewer relapses in the year before treatment start, a higher number of previous DMTs as well as being treated in the Eastern region of Switzerland were associated with a higher probability of treatment with dimethyl fumarate (Supplementary Table 2).

**Table 1:** Baseline characteristics before and after matching

Variable	Unmatched			Matched		
	Fingolimod	Dimethyl fumarate	SMD	Fingolimod	Dimethyl fumarate	SMD
*n*	1075	1038		961 (89%^a^)	961 (93%^a^)	
Age, mean, years (SD)	39.5 (11.5)	40.6 (11.6	0.09	40.2 (11.3)	40.4 (11.5)	0.02
Gender (female, %)	733 (68%)	753 (72%)	0.10	672 (70%)	689 (72%)	0.01
Disease duration, mean, years (SD)	7.9 (8.4)	8.4 (8.0)	0.07	8.1 (8.4)	8.3 (8.0)	0.02
EDSS, median (IQR)	2.0 (1.5–3.0)	2.0 (1.5–3.0)	0.03	2.0 (1.5–3.0)	2.0 (1.5–3.0)	0.01
Preceding treatment, *n* (%)						
Naïve	501 (47%)	393 (38%)		414 (43%)	386 (40%)	
IFN beta 1a i.m.	102 (10%)	133 (13%)		100 (10%)	116 (12%)	
IFN beta 1a s.c.	128 (12%)	151 (15%)		127 (13%)	131 (14%)	
PEG-IFN beta 1a s.c.	11 (1%)	6 (< 1%)		8 (< 1%)	6 (< 1%)	
IFN beta 1b s.c.	84 (8%)	98 (9%)		80 (8%)	88 (9%)	
Glatiramer acetate	90 (8%)	102 (10%)		85 (9%)	88 (9%)	
Teriflunomide	25 (2%)	35 (3%)		24 (3%)	32 (3%)	
Daclizumab	4 (< 1%)	2 (< 1%)		2 (< 1%)	2 (< 1%)	
Ocrelizumab	1 (< 1%)	0		0	0	
Natalizumab	129 (12%)	151 (15%)		121 (13%)	112 (12%)	
Number of relapses in the year before baseline, mean (SD)	0.6 (0.9)	0.5 (0.8)	0.10	0.6 (0.8)	0.6 (0.8)	0.01
Follow up, median, years (IQR)	1.8 (0.9–2.8)	1.8 (0.8–2.8)		0.9 (0.8–1.8)^b^	0.9 (0.8–1.8)^b^	

*SMD* standardized mean difference (> 0.1 is considered as indication of relevant imbalance); *SD* standard deviation; *EDSS* expanded disability status scale; *IQR* interquartile range; *IFN* interferon; *DMT* disease modifying treatment

^a^Proportion of patients retained after matching

^b^After pairwise censoring

The propensity-score matching procedure for the primary analysis retained 961 patients on dimethyl fumarate (93%) and 961 on fingolimod (89%). The matching procedure significantly improved the overall balance as indicated by the baseline characteristics and distribution of the propensity scores (Table 1, Supplementary Figure).

**Fig. 2:**
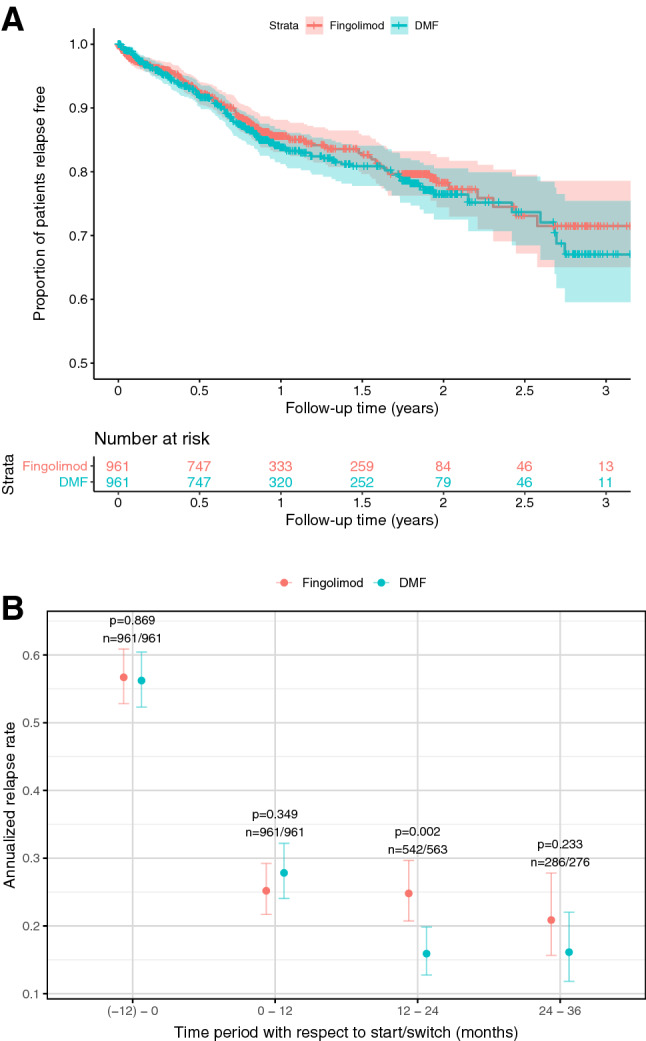
Relapse outcomes. **a** Proportion of patients free from relapses. Shaded areas represent 95% confidence intervals. **b** Annualised relapse rates at baseline, 12, 24 and 36 months. Error bars represent 95% confidence intervals. *HR* hazard ratio, *EDSS* Expanded Disability Status Scale, *DMT* disease modifying treatment

**Fig. 3:**
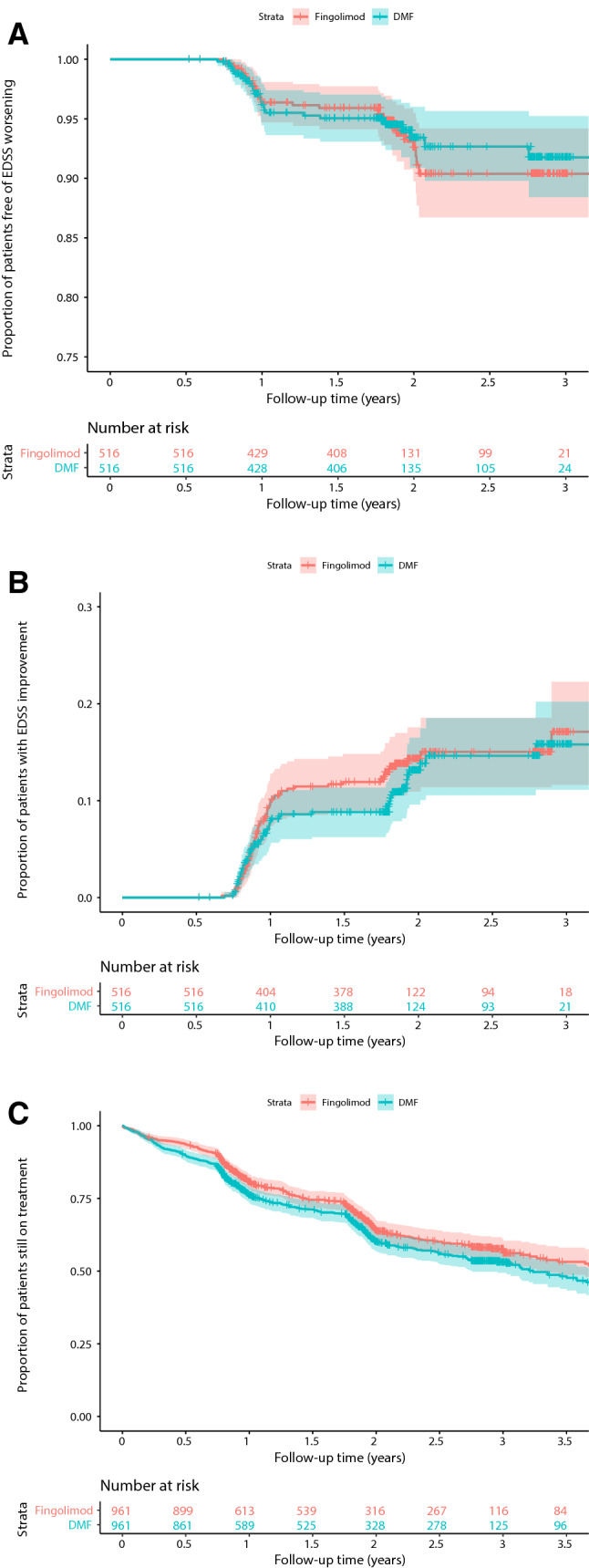
Disability outcomes and treatment persistence. **a** Proportion of patients free from disability worsening. **b** Proportion of patients with disability improvement. **c** Proportion of patients still on treatment. Shaded areas represent 95% confidence intervals

The median follow-up time after pairwise censoring was 0.9 years (interquartile range 0.8–1.8). We did not observe any difference in the time to first relapse between the dimethyl fumarate- and fingolimod-treated groups (HR 1.1, 95%CI 0.9–1.4, *p* = 0.41, Fig. 2a). Annualised relapse rates dropped in both the dimethyl fumarate and the fingolimod group without a significant between-group difference (incident rate ratio 1.0, 95%CI 0.9–1.5, *p* = 0.18, Fig. 2b). Moreover, we did not observe any differences regarding the hazard of one-year confirmed disability worsening (HR 0.9, 95%CI 0.6–1.6, *p* = 0.80, Fig. 3a) or of 1-year confirmed disability improvement (HR 0.9, 95%CI 0.6–1.2, *p* = 0.40; Fig. 3b) in matched patients with sufficient follow-up (dimethyl fumarate, *n* = 516; fingolimod, *n* = 516).

The secondary analyses in treatment-naïve patients and treatment switchers did also not show any differences between the groups, except for a higher chance of fingolimod-treated patients to experience confirmed disability improvement in the treatment-switchers (Table 2).

**Table 2:** Secondary analyses

	^a^HR (95% CI) ^b^IRR (95% CI)	*p* value
(A) First-line therapy, *n* = 786, for relapse outcomes, *n* = 368 for disability outcomes		
Free from relapses^a^	0.7 (0.5–1.1)	0.11
ARR^b^	0.8 (0.6–1.9)	0.54
Free from disability worsening^a^	2.0 (0.6–6.8)	0.25
Disability improvement^a^	1.1 (0.7–1.9)	0.56
(B) Second-line therapy, *n* = 588 for relapse outcomes, *n* = 350 disability outcomes		
Free from relapses^a^	1.0 (0.7–1.5)	0.83
ARR^b^	1.0 (0.5–1.5)	0.79
Free from disability worsening^a^	0.8 (0.4–1.7)	0.53
Disability improvement^a^	0.4 (0.2–1.0)	0.04

Comparison of dimethyl fumarate vs. fingolimod (reference)

*HR* hazard ratio, *IRR* incident rate ratio, *CI* confidence interval, *ARR* annualised relapse rate

^a^For the proportions of patients without relapse, without disability progression and with disability improvement, Cox marginal proportional hazards models are used

^b^ARRs are compared with negative binomial models

The sensitivity analyses confirmed the results of the primary analysis to full extent (Supplementary Table 3).

After 1 year, the proportion of patients continuing on treatment with fingolimod was higher than on dimethyl fumarate (87%, 95%CI 85–90% vs. 81%, 95%CI 76–83%, *p* = 0.002), but the difference was no longer statistically significant when considering the entire available follow-up time (HR 1.2, 95%CI 1.0–1.3, *p* = 0.06, Fig. 3c).

When analysing follow-up treatment after discontinuation of one of the drugs, we found that most patients switched from dimethyl fumarate to fingolimod and vice versa, followed by natalizumab (Table 3). Among the patients switched to natalizumab about two-thirds had experienced a relapse in the year prior to the switch and the average relapse rate was the highest in this group.

**Table 3:** Treatments after discontinuation

	*n* (%^a^)	Patients with ≥ 1 relapse, *n* (%^b^)	ARR (SD)
Fingolimod			
Dimethyl fumarate	35 (27%)	11 (31%)	0.5 (1.0)
Natalizumab	32 (25%)	22 (69%)	0.9 (0.9)
Ocrelizumab	13 (10%)	5 (39%)	0.5 (0.7)
Teriflunomide	12 (9%)	4 (33%)	0.4 (0.5)
Glatiramer acetate	12 (9%)	4 (33%)	0.3 (0.5)
Interferon beta-1a i.m.	9 (7%)	3 (33%)	0.6 (0.9)
Interferon beta-1a s.c.	9 (7%)	0	0
Interferon beta-1b s.c.	3 (2%)	0	0
Daclizumab beta	3 (2%)	0	0
Dimethyl fumarate			
Fingolimod	69 (38%)	27 (39%)	0.5 (0.8)
Natalizumab	38 (21%)	23 (61%)	0.9 (1.1)
Teriflunomide	15 (8%)	3 (20%)	0.3 (0.6)
Glatiramer acetate	15 (8%)	5 (33%)	0.5 (0.8)
Ocrelizumab	11 (6%)	4 (36%)	0.5 (0.9)
Interferon beta-1a i.m.	9 (5%)	2 (22%)	0.2 (0.5)
PEG-Interferon beta-1a s.c.	8 (4%)	2 (25%)	0.2 (0.4)
Interferon beta-1a s.c.	7 (4%)	1 (14%)	0.1 (0.4)
Interferon beta-1b s.c.	6 (3%)	1 (16%)	0.2 (0.4)
Daclizumab beta	2 (1%)	0	0

*ARR* annualised relapse rate

^a^Percentage of all patients discontinuing therapy

^b^Percentage of patients switching to respective drug

## Discussion

In this observational, propensity-score matched analysis of a large MS cohort from Switzerland, we found that dimethyl fumarate and fingolimod had comparable effects on relapse activity and disability outcomes. This finding was consistent for treatment-naïve patients and those switching from another DMT. Treatment persistence was lower with dimethyl fumarate after 1 year but not significantly different over the whole follow-up period.

The pivotal phase III trials of dimethyl fumarate, DEFINE and CONFIRM, found a significant reduction in ARR by about 50% compared with placebo and by 29% compared with glatiramer acetate, respectively [6, 7]. In addition, the placebo-controlled DEFINE trial showed a 38% lower risk of 12-week confirmed disability progression for dimethyl fumarate, which could not be seen in comparison with glatiramer acetate. The efficacy of fingolimod was evaluated in three large phase III trials [3–5]. In the placebo controlled studies (FREEDOMS/FREEDOMS II), fingolimod was shown to be superior compared with placebo, reducing the ARR by 54% and 48%, respectively. The FREEDOMS trial also demonstrated a reduced risk of 6-months confirmed disability progression by 37% under fingolimod and the active-controlled TRANSFORMS trial could show a superior efficacy of fingolimod over interferon beta-1a with respect to relapse rates [3].

As no data from a randomised head-to-head trial between dimethyl fumarate and fingolimod was available, an attempt to assess the comparative efficacy of the two drugs was made by analysing pooled data from the CONFIRM, DEFINE and FREEDOMS/FREEDOMS II studies with a matching-adjusted, indirect approach [8]. This analysis found a comparable efficacy for both compounds on relapse activity and disability progression. However, the study was subject to unknown or unmeasured confounders similar to observational studies and also of limited generalisability.

An observational study from a single, large academic centre followed 358 patients treated with dimethyl fumarate and 317 treated with fingolimod over the period of 12 months [12]. Interestingly, the study also included a proportion of patients with progressive disease courses (26% and 12%, respectively) and in contrast to our study, rather few patients were on first-line dimethyl-fumarate (8%) and fingolimod treatment (5%), respectively. Both 1:1 propensity score-matching and Average Treatment effect on the Treated (ATT)-weighting based on the propensity score were used to mitigate indication bias. The study found no significant differences in ARR between the treatment groups and similar ARRs compared to phase III therapeutic trials. Moreover, measures of neurologic impairment, namely the Timed 25-Foot Walk and the 9-Hole Peg Test showed no difference between the groups. Also, no difference in a composite measure of MRI activity or new T2 lesions was found, although patients treated with dimethyl fumarate had a higher risk of developing gadolinium enhancing lesions by 12 months. Of note, patients treated with dimethyl fumarate experienced first relapses earlier than fingolimod patients and were more likely to discontinue treatment due to intolerability. A 24-months follow-up study from the same centre largely confirmed these results [13].

Another single centre study included 613 patients (dimethyl fumarate, *n* = 342, fingolimod *n* = 271) followed for up to 2 years, of which approximately 25% were treatment naïve and 17% had a progressive disease course [14]. The analysis was performed using logistic regression, propensity-score matching and ATT-weighting and found no difference between dimethyl fumarate and fingolimod on a composite outcome including clinical relapses and MRI activity, but increased odds of treatment discontinuation for dimethyl fumarate.

Recently, a pooled analysis of data from these two centres confirmed the results of the previously published studies showing comparable clinical and radiographic effectiveness profiles of both drugs, but demonstrating higher odds of dimethyl fumarate discontinuation compared with fingolimod [15].

A multicentre study from Italy analysed the effect of both compounds on No Evident Disease Activity (NEDA)-3 status, defined as being free from relapses, disability worsening and radiologic activity [17]. The study analysed 550 propensity-score matched patients with relapsing–remitting MS, of which 179 were treatment naïve and 389 switched from platform injectable drugs with a median follow-up of 18 months. While the analysis revealed a similar effectiveness of dimethyl fumarate and fingolimod on NEDA-3 and its subcomponents in the whole cohort, the subgroup analysis of treatment switchers found a 43% increased likelihood of achieving NEDA-3 status in the short-term follow-up for fingolimod compared with dimethyl fumarate driven by fewer relapses and less 6 months-confirmed disability progression.

The largest observational study to date originated from the MSBase registry and analysed 504 propensity-score matched patients on DMF and 1825 on fingolimod, with a mean ARR prior to inclusion of 0.8 and 0.9, respectively and a median follow-up of 1.3 years [16]. The authors demonstrated a lower hazard for relapses under fingolimod compared to DMF, but no difference between the two drugs in terms of confirmed disability worsening or improvement.

In our study, we found a comparable effectiveness of dimethyl fumarate and fingolimod on relapse activity in our analysis of 1922 propensity-score matched patients. In contrast to the study by Prosperini et al., our results were consistent for all subgroups irrespective of previous disease modifying treatment [17]. As the mean pre-switch ARR was substantially higher in this study compared to our subgroup of treatment switchers (1.3 vs. 0.6) one could speculate that fingolimod might have an advantage over DMF in patients with a higher disease activity than in our cohort. We also cannot exclude that unavailability of MRI data for the matching procedure has confounded our results to a certain degree. However, the absolute risk reduction for relapses reported in the study by Prosperini et al. [17] was only of a moderate magnitude (72% vs. 68%). Similarly, the study from MSBase resembling our cohort more closely in terms of baseline characteristics, showed only a very modest absolute risk reduction of approximately 0.06 relapses per year [16].

When analysing 1 year-confirmed disability worsening, we were not able to detect a difference between the two groups, in line with the analysis from MSBase [16]. While disability worsening measured by the EDSS was not analysed in the studies performed by Hersh et al., and Vollmer et al., [13–15] the Italian study found a lower risk of 6-months confirmed disability worsening for fingolimod vs. dimethyl fumarate in the whole cohort as well as in treatment switchers [17]. This discrepancy could be explained by our definition of disability worsening, requiring confirmation after at least 1 year, which has been shown to be less sensitive but more specific than shorter confirmation intervals [20].

We found that treatment discontinuation during the first year was slightly more frequent in the dimethyl fumarate than in the fingolimod group (19% vs. 13%) confirming findings from the pivotal RCTs as well as the previously mentioned observational studies. Unfortunately, information about reasons for treatment discontinuation were not documented in our registry. Of note, the difference between the groups diminished after the first year of treatment. Hence, we can assume that lack of tolerability of dimethyl fumarate early after treatment initiation was the strongest driver for discontinuation, as has been shown in previous studies [16, 17].

The main limitations of our study apart from the observational, non-randomised study design are the lack of MRI data and the relatively short median follow-up time of 1 year after pairwise censoring. Although MRI scans are routinely performed as required by clinical practice before treatment initiation, on-treatment and before planned treatment switch, MRI parameters are not recorded in the SVK registry in detail, so that we could not use MRI metrics in the matching process or as an outcome variable. As inclusion of MRI data is beyond the scope of the SVK registry, we plan to utilise the Swiss Multiple Sclerosis Cohort study (SMSC), which is currently performed at tertiary Swiss MS centres for future analysis of systematically acquired clinical data, standardised MRI and body fluid biomarkers (such as serum Neurofilament light chain), once patient numbers and follow-up time are sufficient [23]. In addition, the exclusion of patients without at least one follow-up visit may have introduced a bias towards equivalence between the two treatment groups. However, the number of patients excluded because of insufficient follow-up was relatively low (804; 15%), which argues against a pronounced effect. To mitigate the known treatment indication bias, we employed propensity score-matching. Unlike randomisation, this technique cannot eliminate bias due to unknown or unmeasurable confounders. However, this is unlikely to have a substantial effect on our overall conclusions, as sensitivity analyses with varying inclusion criteria confirmed the results of the primary analysis. We ensured comparison within the same treatment era by excluding patients who commenced fingolimod treatment before dimethyl fumarate was available. Pairwise censoring was applied to control for attrition bias. As yearly visits were mandatory, adjustment for reporting bias by taking-into account the frequency of clinical follow-up was not necessary. Generalisability of our study results was maximised by inclusion of a broad spectrum of patients.

In conclusion, our observational study adds further evidence to the comparable effectiveness of dimethyl fumarate and fingolimod in a real-world setting. Further analyses, ideally including MRI data are needed to assess the comparative long-term effectiveness of the two compounds.

## Electronic supplementary material

Below is the link to the electronic supplementary material.

Supplementary material 1 (PDF 122 kb)

## Data Availability

The data that support the findings of this study are available from the corresponding author upon reasonable request.
