# Supplementary material for: Comparative analysis of dimethyl fumarate and fingolimod in relapsing–remitting multiple sclerosis
Source: J Neurol. 2020 Sep 24;268(3):941–9. doi: 10.1007/s00415-020-10226-6 (PMC7914245; doi:10.1007/s00415-020-10226-6)

Supplementary Table 1: Baseline characteristics of the dataset used to determine disability progression.

**Variable**	**Unmatched**			**Matched**		
	**Fingolimod**	**Dimethyl fumarate**	**SMD**	**Fingolimod**	**Dimethyl fumarate**	**SMD**
n	625	597		516 (83%[†])	516 (86%[†])	
Age, mean, years (SD)	39.2 (10.9)	41.6 (11.4)	0.21	40.3 (11.0)	41.0 (11.3)	0.07
Gender (female, %)	424 (68%)	431 (72%)	0.10	361 (70%)	365 (71%)	0.02
Disease duration, mean, years (SD)	7.9 (8.2)	8.9 (8.0)	0.13	8.4 (8.5)	8.5 (7.7)	0.02
EDSS, median (IQR)	2.0 (1.5-3.0)	2.0 (1.5-3.0)	0.005	2.0 (1.5-3.0)	2.0 (1.5-3.0)	0.02
Preceding treatment, n (%)						
Naïve	280 (45%)	196 (32%)		199 (39%)	189 (37%)	
IFN beta 1a i.m.	62 (10%)	97 (16%)		59 (11%)	72 (14%)	
IFN beta 1a s.c.	72 (12%)	92 (15%)		71 (14%)	72 (14%)	
IFN beta 1b s.c.	55 (9%)	68 (11%)		53 (10%)	57 (11%)	
Glatiramer acetate	56 (9%)	59 (10%)		52 (10%)	49 (10%)	
Teriflunomide	14 (2%)	21 (4%)		14 (3%)	15 (3%)	
Natalizumab	83 (13%)	62 (10%)		66 (13%)	60 (12%)	

Number of relapses in the year before baseline, mean (SD)	0.6 (0.8)	0.5 (0.7)	0.11	0.6 (0.8)	0.5 (0.8)	0.06

†proportion of retained patients after matching

SMD = standardized mean difference; SD = standard deviation; EDSS = expanded disability status scale; IQR = interquartile range; IFN = interferon; DMT = disease modifying treatment

Supplementary table 2: Logistic model used to determine the propensity scores

Independent variable	Estimate (SE)	p-value
Gender, male	-0.0044 (0.1809)	0.9805
Age	0.0053 (0.0088)	0.5420
Disease duration	0.0001 (0.0138)	0.9966
EDSS	0.0157 (0.1291)	0.9033
EDSS increase in last year	-0.1836 (0.2166)	0.3968
Preceding relapse rate	-0.1715 (0.0762)	0.0245
Visual FSS	-0.0129 (0.1159)	0.9112
Brainstem FSS	0.1111(0.1381)	0.4209
Pyramidal FSS	-0.0404 (0.1136)	0.7220
Cerebellar FSS	-0.1762 (0.1282)	0.1692
Sensory FSS	0.0833 (0.1014)	0.4117
Bowel and bladder FSS	0.1254 (0.1257)	0.3186
Cerebral FSS	-0.1821 (0.1021)	0.0746
Time on previous DMT	-0.0330 (0.0233)	0.1568
Number of previous DMT	0.4208 (0.1966)	0.0350
Time since last relapse	0.0269 (0.0271)	0.3204
North-Western region[†]	0.4307 (0.3344)	0.1977
Northern region[†]	-0.0088 (0.3314)	0.9789
Central region[†]	0.2626 (0.3512)	0.4546
Eastern region[†]	0.8625 (0.3067)	0.0050

Southern region[†]	0.0732 (0.4798)	0.8788

Propensity for treatment with dimethyl fumarate vs. fingolimod (reference) at baseline.

SE = Standard error, EDSS = Expanded disability status scale, FS = Functional System Score, DMT = Disease modifying treatment

[†]The South-Western region is used as a reference.

Supplementary Table 3: Sensitivity analyses

**1) Matching with a calliper of 0.1**		
	[†]**HR (95% CI)** [‡]**IRR (95% CI)**	**p-value**
Free from relapses[†]	1.1 (0.9-1.4)	0.53
ARR[‡]	1.1 (0.9-1.4)	0.32
Free from disability worsening[†]	1.0 (0.6-1.7)	0.99
Disability improvement[†]	0.9 (0.6-1.3)	0.80
**1) No pairwise censoring**		
Free from relapses[†]	1.1 (0.9-1.4)	0.37
ARR[‡]	1.1 (0.9-1.3)	0.37
Free from disability worsening[†]	0.8 (0.5-1.2)	0.22
Disability improvement[†]	0.8 (0.6-1.2)	0.26
**3) Including patients irrespective of treatment start**		
Free from relapses[†]	0.9 (0.7-1.1)	0.24
ARR[‡]	1.2 (1.0-1.4)	0.11
Free from disability worsening[†]	1.0 (0.6-1.5)	0.93
Disability improvement[†]	0.9 (0.6-1.2)	0.29

Comparison of dimethyl fumarate vs. fingolimod (reference).

[†]For the proportions of patients without relapse, without disability progression and with disability improvement, Cox marginal proportional hazards models are used.

[‡]ARRs are compared with negative binomial models.

HR = hazard ratio, IRR = incident rate ratio; CI = confidence interval; ARR = annualised relapse rate

Supplementary figure. Distribution of propensity scores before and after matching

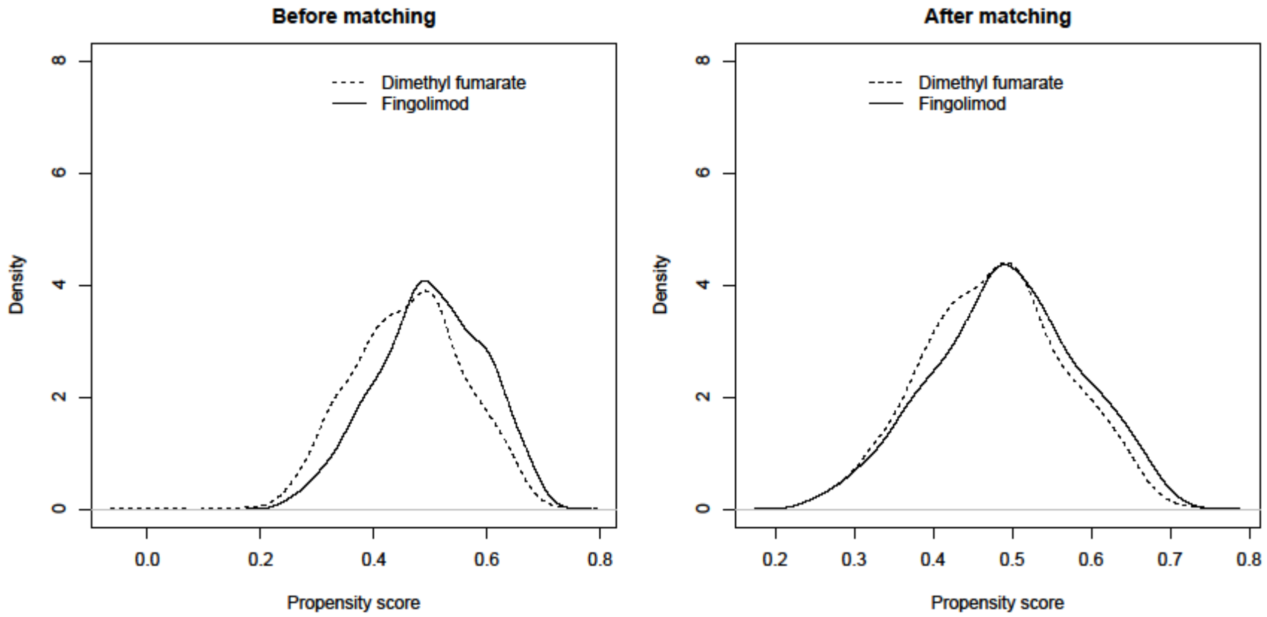

Supplement: Supplementary file 1 — Supplementary material 1 (PDF 122 kb) [file 415_2020_10226_MOESM1_ESM.pdf]
